# Household Hints and Recipes

**Published:** 1883-01

**Authors:** 


### Household Hints & Recipes.

CEMENT FOR AMBER.—The *Canadian Pharmaceutical Journal* states that amber may be cemented by moistening the surfaces with solution of potassa and pressing them together.

TOOTHACHE REMEDY.—The following is recommended;

Tinct. iodine, 10 drops.

Carbolic acid (dil.), ½ drachm.

Chloroform, ½ drachm.

Mix, and apply to the gums.

SHOE VARNISH.—

Alcohol, 2 pints.
Resin, 3 ounces.
Shellac, 4½ ounces.
Lampblack, 1 ounce.
Oil of sassafras, 1 ounce.

Mix. The varnish is to be applied with a small brush.

PERFUME FOR VIOLET POWDER.—

Bergamot oil, 20 parts.
Lemon oil, 20 parts.
Clove oil, 10 parts.
Neroli, 10 parts.

Use equal parts of powdered orrisroot and starch, and add 1 drachm of this to each pound of the powder.

CHILBLAINS.—The following formula for the relief of itching and irritation is recommended by Dr. Hildreth, in the *Medical Brief:*

Sulphurous acid, 1 ounce.
Glycerine, 1 ounce.
Distilled water, 2 ounces.

Apply night and morning.

Another formula is given by Kilner:

White wax, 2 drachms.
Spermaceti, 2 drachms.
Balsam Peru, 1 drachm.
Olive oil, 3 ounces.
Muriatic acid, 2 drachms.
Water, 6 drachms.

Make a plaster, and apply.

HOW TO CLEAN PENS.—A writer in a German paper states that it is a custom in offices in that country to have a sliced potato on the desk in commercial houses. He does not state whether the esculent should be raw or not, but as it is not for the purpose of making kartoffelsalat, it is probably raw. It is used to clean steel pens, and generally acts as a pen-wiper. It removes all ink-crust, and gives a peculiarly smooth flow to the ink. He also states that the Hamburg clerks pass new pens two or three times through a gas flame, and then the ink will flow freely.—*Southern Medical Record.*

FLORIDA WATER.—

Oil of lavender, 4 ounces.
Oil of bergamot, 4 ounces.
Oil of cinnamon, 2 drachms.
Oil of cloves, 1 drachm.
Oil of neroli, 2 drachms.
Pure musk, 4 grains.
Cologne spirits, 95 per cent., 1 gallon.

Macerate fifteen days and filter through paper. We are not able to give a formula for Fellows' Syrup of Hypophosphites Comp., but we here republish the formula of the new U. S Pharmacopœia. All parts by weight.

FOR COATING INSECTS, LEAVES, AND FLOWERS WITH COPPER.—The proper preparation of the articles to be coated is more important than the special form of battery to be used. A leaf, for instance, should be carefully dried between blotters in a warm place. Next it should be coated with some thin varnish, or dipped into melted wax, and then brushed over with "electroplater's plumbago" in order to give it a surface capable of conducting electricity. A very fine copper wire is then attached and made to actually touch the plumbago coating. The leaf may now be suspended in a bath containing

Sulphate of copper ("blue vitriol") 4 ounces.
Sulphuric acid, ¼ ounce.
Water, cistern, 1 quart.

Into this liquid is placed a small "porous cell" (a flower pot with the bottom corked will answer). The porous cell is partly filled with a mixture of

Sulphuric acid, 1 ounce.
Rain water, 12 ounces.

Into this liquid insert a small rod of cast zinc in such a manner as to expose less surface to the action of the acid than the surface of the leaf to be coated. Now connect the upper part of the zinc rod with the fine wire from the leaf. Copper will be slowly deposited. If done too fast the coating will not be perfect.

